# Monitoring Health and Well-Being in Emerging Adults: Protocol for a Pilot Longitudinal Cohort Study

**DOI:** 10.2196/16108

**Published:** 2020-04-23

**Authors:** Reidar P Lystad, Diana Fajardo Pulido, Lorna Peters, Melissa Johnstone, Louise A Ellis, Jeffrey Braithwaite, Viviana Wuthrich, Janaki Amin, Cate M Cameron, Rebecca J Mitchell

**Affiliations:** 1 Australian Institute of Health Innovation Macquarie University Sydney Australia; 2 Centre for Emotional Health Department of Psychology Macquarie University Sydney Australia; 3 Department of Educational Studies Macquarie University Sydney Australia; 4 Department of Health Systems and Populations Macquarie University Sydney Australia; 5 Jamieson Trauma Institute Metro North Hospital and Health Service Queensland Health Brisbane Australia

**Keywords:** young adults, emerging adulthood, health, well-being, health-related quality of life

## Abstract

**Background:**

Emerging adulthood is a unique segment of an individual’s life course. The defining features of this transitional period include identity exploration, instability, future possibilities, self-focus, and feeling in-between adolescence and adulthood, all of which are thought to affect quality of life, health, and well-being. A longitudinal cohort study with a comprehensive set of measures would be a unique and valuable resource for improving the understanding of the multi-faceted elements and unique challenges that contribute to the health and well-being of emerging adults.

**Objective:**

The main aim of this pilot study is to evaluate the feasibility and acceptability of recruiting university graduates to establish a longitudinal cohort study to inform our understanding of emerging adulthood.

**Methods:**

This is a pilot longitudinal cohort study of Australian university graduates. It will involve collecting information via online surveys (baseline and 12-month follow-up) and data linkage with health records. Recruitment, response, and retention rates will be calculated. Descriptive analysis of the representativeness of recruited participants and completeness of survey responses will be conducted.

**Results:**

Participant recruitment was completed in October 2018, and data collection for the baseline and follow-up surveys was completed in November 2019. As of April 2020, the process of acquiring health records from administrative data collections has commenced.

**Conclusions:**

The findings from this pilot study will identify areas for improvement and inform the development of a future longitudinal cohort study of emerging adults.

**Trial Registration:**

Australian New Zealand Clinical Trials Registry ACTRN12618001364268; https://tinyurl.com/teec8wh

**International Registered Report Identifier (IRRID):**

DERR1-10.2196/16108

## Introduction

Throughout young people’s lives, there are many events and factors that can affect their life course. Emerging adulthood is the transitional period from late teens through to the late twenties and is characterized by a higher degree of diversity, instability, and uncertainty [1]. The defining features of emerging adulthood include identity exploration (ie, exploring available options for life especially in love and work); instability (ie, being subject to numerous changes and shifting choices); future possibilities (ie, multiple available options where different futures remain possible); self-focus (ie, focus on forming oneself); and feeling in-between (ie, neither an adolescent nor an adult). Demographic norms change considerably during emerging adulthood, especially in terms of residential status and school attendance. In their late teens, most people live with one or more parents and attend school; whereas most people in their thirties work full-time, live independently, and cohabitate with a romantic partner. These features are thought to impact the quality of life and well-being of emerging adults [2].

Transitioning from education to work life can be particularly challenging for emerging adults [3]. While the experience of tertiary education gives the opportunity to explore different identities and lifestyles, work is often more salient in shaping one’s identity because of its central role in adult life [2]. Work can be pivotal for long-term prospects, such as acquiring financial independency, career, marriage or partnership, and parenthood [2]. Difficulties in transitioning between education and work life can negatively affect the health and well-being of emerging adults, and unsuccessful transitions can lead to mental health problems later in life [4,5].

The sense of instability, uncertainty, and multitude of future possibilities can negatively impact physical health, health-related quality of life (HRQOL), and well-being of emerging adults [6]. HRQOL is a multi-dimensional concept that purports to quantify the relationships between physical and mental health status and quality of life over time [7]. Many popular HRQOL metrics typically measure self-perceived health status [8]. A related concept is well-being, which evaluates the positive aspects of an individual’s life, including life satisfaction and positive emotions [9]. Both HRQOL and well-being have been used to measure the impact of illness and disability on the quality of life of emerging adults. For instance, Pons-Villanueva et al [10] found that university graduates who had been involved in a motor vehicle crash had poorer HRQOL four years post crash. Van Oostrom et al [11] reported that adults who had adopted an active lifestyle experienced better HRQOL over time. Further, Buhl [3] identified that emerging adults who did not go to university reported poorer parent-child relationships compared to those who transitioned from university to work life.

Although several studies have investigated aspects of HRQOL and well-being in emerging adults, more information is needed to better understand these relationships. A more comprehensive view of the multi-faceted elements and unique challenges that contribute to the health and well-being of emerging adults, including education, employment, lifestyle, HRQOL, well-being, social support, life events, carer responsibilities, and use of social media technology is needed. Thus, conducting a large, longitudinal cohort study with a comprehensive set of measures would be a unique and valuable resource for improving our understanding of education, health, and lifestyle factors and their impact on resilience, career trajectories, and lived experiences over a unique segment of an individual’s life course.

This pilot cohort study aims to establish the feasibility and acceptability of recruiting university graduates to establish a longitudinal cohort study to inform our understanding of emerging adulthood. Specifically, the study will evaluate: (1) the feasibility of our research methods to recruit university graduates at one large Australian university, including determination of opt-in and opt-out rates for data linkage of health records and survey responses; (2) the representativeness of recruited participants; (3) our ability to obtain baseline survey data, including completion of individual survey instruments; (4) our ability to retain participants and collect follow-up survey data 12 months post baseline, including completion of individual survey instruments; (5) and identify areas for improvement for future studies.

## Methods

### Registration

This study was registered with the Australian New Zealand Clinical Trials Registry (ACTRN) on August 14, 2018 (ACTRN12618001364268).

### Design

**Figure 1:**
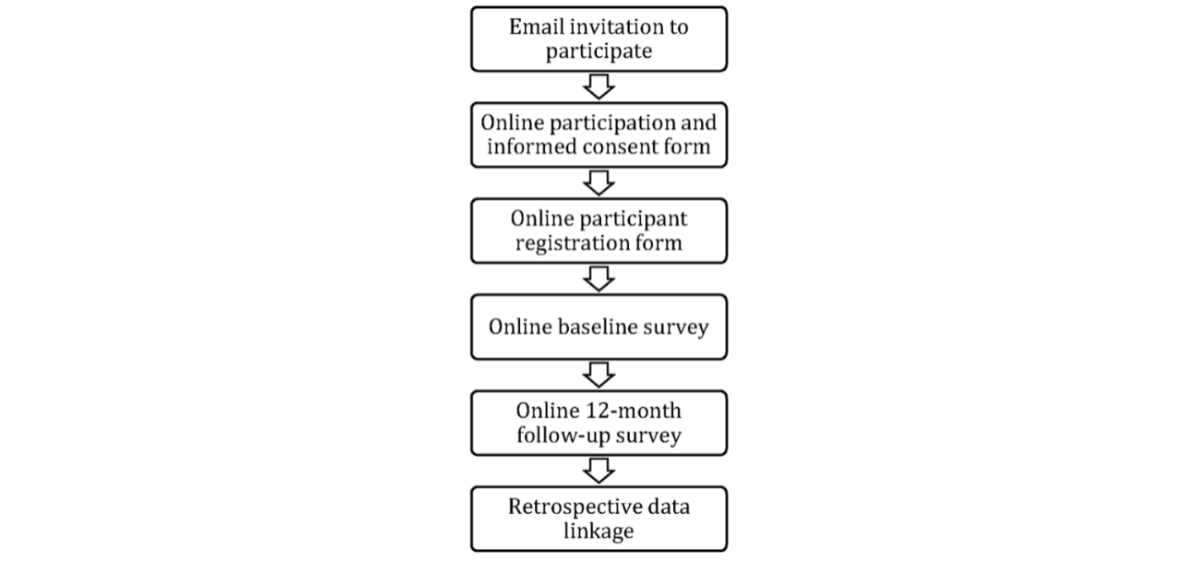
Flowchart of the study design.

This is a pilot longitudinal cohort study of Macquarie University graduates. It involves collecting information via online surveys (ie, baseline and 12-month follow-up) and data linkage with health records. A flowchart of the study design is depicted in Figure 1.

### Recruitment

All students graduating from Macquarie University in 2018 are eligible to participate in the study. Macquarie University is a public university located in Sydney, Australia. The university has five faculties (ie, Faculty of Arts, Faculty of Business and Economics, Faculty of Human Sciences, Faculty of Medicine and Health Sciences, and Faculty of Science and Engineering), which collectively host approximately 45,000 students, including 33,000 undergraduate students, 9000 postgraduate students, and 1500 higher-degree research students. Each year, approximately 7000 students graduate with an undergraduate or postgraduate degree.

Invitations to participate in this study will be distributed to the graduates via email from the Macquarie University Graduation Office in conjunction with the fall and spring graduation ceremonies (ie, April 12-27, 2018 and September 19-28, 2018). The initial email invitation will be sent out during the graduation ceremony period, while three reminder emails will be sent out during the following 4-6 weeks. The email invitations include a brief description of the purpose of the study, what participation will involve, and a link to the *Macquarie University – Monitoring of Injury and Psychosocial Health Outcomes, Career Trajectories and Continuing Education, Lived Experiences, and Social Connectedness* (MQ-MINDS) project website with a full Participant Information and Consent Form. The Participant Information and Consent Form contains details about the purpose of the study, what participation will involve, a description of benefits and risks of taking part in the study, confidentiality and privacy arrangements, funding for the study, and consent to participate.

Participants will be given the opportunity to opt out of having their survey responses linked to their health records (ie, ambulance, emergency department, hospitalization, cancer registry, and mortality records). Participants are then directed to an online participant registration form that securely records their personally identifiable information, including their name, residential address, mobile phone number, and email address. Upon completing the online registration form, participants will receive an email with an individualized link to the baseline survey.

### Data Collection

Data will be collected through online surveys and, for those that did not opt out, health record linkage. The online surveys will be administered online via the Qualtrics XM Platform (Qualtrics International Inc) at baseline and at 12 months post baseline. The baseline and 12-month follow-up surveys will comprise the same battery of validated questionnaires and instruments designed to capture data regarding: sociodemographics, education, employment, job satisfaction, mentoring, self-perceived health status, work-life balance, connectedness, resilience, injury, risk behaviors, life events, as well as social media and technology use. It will take approximately 25 minutes to complete the online survey.

### Survey Instrument

An overview of the domains and specific questionnaires included in the survey is provided in Table 1. Because the target population is a subset of the general Australian population, the survey is comprised of instruments that are designed or adapted for use in the Australian population, whenever possible. This will facilitate more direct comparison of the data collected in this study with existing normative data from the general Australian population.

**Table 1:** Overview of survey domains and measures.

Domain	Measures
Sociodemographics	Standardized questions about gender, sexual orientation, height, weight, ethnicity, language, marital status, house tenure, and income
Tertiary education	Questions about previous academic qualifications and current enrollment in tertiary education
Employment	Questions about occupation, employment status, job satisfaction, job barriers, and future employment goals Questions about career mentoring Role Balance Scale
Lifestyle	Questions about physical activity and sedentary behavior
Health	EuroQoL 5-dimension Short Form Health Survey Kessler Psychological Distress Scale Social Interaction Anxiety Scale General Anxiety Disorder scale Questions about smoking, alcohol consumption, drug use, and sexual behavior
Social support	Multidimensional Scale of Perceived Social Support Questions about social participation (eg, community, church, or self-help groups)
Life events	Social Readjustment Rating Scale Brief Resilience Scale
Carer activities	Questions about carer responsibilities and activities
Social media and technology	Questions about access to the internet and devices used (eg, laptop, mobile phone, tablet) Questions about use of social networking sites (eg, Facebook, Twitter, Snapchat) Questions about experiences with using social media

#### Sociodemographics

For the sociodemographic domain, questions about gender, sexual orientation, ethnicity, language, marital status, living arrangement, and household income are derived from the New South Wales Population Health Survey [12].

#### Tertiary Education

The education domain includes questions about previous academic qualifications, level of previous academic degrees, and current academic programs.

#### Employment

The employment domain is comprised of three subdomains: general questions about current employment, work-life balance, and career mentoring. In regard to current employment, questions about occupation, employment status, job satisfaction, and future employment goals are adapted from the Australian Workplace Relations study [13]. Job satisfaction is assessed on a 7-point Likert scale ranging from “extremely satisfied” to “extremely dissatisfied,” within seven different perceived aspects of the current job (ie, flexibility, decision making, autonomy, salary, job security, job content, and working conditions) and an overall question: “how satisfied are you with your current job?”. Information on work-life balance is recorded using the Role Balance Scale (RBS) [14]. The RBS consists of eight items that evaluate the engagement of participants across different roles and the ability to incorporate the newly emerging roles within their life. The first five statements focus on the balance and enjoyment across different roles, and the distribution of importance between roles and overall satisfaction. The last three statements indicate the self-perceived importance of each role in the participants' lives. Information about the perceived benefits and potential role of mentors in the participants’ career will be collected using an adapted instrument developed by DeCastro et al [15]. There are nine items that consider different aspects of mentoring relationships (eg, improvement of job performance; mentor perceived as a role model; increased social network; advise to further develop professional career; resources to develop professional career; advise in keeping work-life balance; and develop new knowledge, skills and ethical behavior). These items are assessed on a 7-point Likert scale ranging from “extremely satisfied” to “extremely dissatisfied.”

#### Lifestyle

Questions about physical activity and sedentary behavior are adapted from the New South Wales Population Health Survey [12]. These include questions about time spent walking and frequency; time spent doing moderate, strengthening, and vigorous activities per week; and time spent sleeping, sitting at work, watching television, and using technology devices such as computers, tablets, or smartphones.

#### Health

The health domain comprises several instruments assessing various aspects of physical and mental health and HRQOL. The 12-item Short Form Health Survey (SF-12) provides insight regarding the participant’s physical and mental health measured through eight dimensions (ie, physical functioning, role physical, role emotional, mental health, body pain, general health, vitality, and social functioning) using a 7-point Likert scale [16]. The SF-12 has demonstrated great feasibility in monitoring the health of specific populations [16].

The EuroQoL 5-dimension (EQ-5D) is a widely used instrument to describe and value health. It comprises two parts: a five-item descriptive system and a visual analogue scale [17]. The five items (ie, mobility, self-care, usual activities, pain/discomfort and anxiety/depression) are rated using five levels: “No problems,” “Slight problems,” “Moderate problems,” “Severe problems,” and “Extreme problems.” The final question asks respondents to rate their health on a scale ranging from 0 (ie, “best imaginable health state”) to 100 (ie, “worst imaginable health state”) [18].

The General Anxiety Disorder scale (GAD-7) is a tool with strong validity to identify probable cases of GAD that can be associated with multiple domains of functional impairment and disability days [19]. It consists of seven items that identify symptoms of anxiety (eg, feeling nervous or anxious, being unable to stop or control worrying, worrying too much, having trouble relaxing, being restless, becoming easily annoyed or irritable, and feeling afraid as if something awful might happen) over the past 2 weeks, and rates their severity on a scale from 0 (“Not at all”) to 3 (“Nearly every day”). If applicable, the GAD-7 also includes a question about the respondent’s perceived difficulty in performing daily activities due to these symptoms [19].

The Social Interaction Anxiety Scale (SIAS-6) is an accurate and efficient psychometric instrument that aims to assess social interaction anxiety as the core feature of social anxiety disorder [20]. The instrument is comprised of six statements about meeting and talking to strangers, friends, or members of the opposite sex. Each statement is rated using a 5-point scale, ranging from 0 (“Not at all characteristic or true of me”) to 4 (“Extremely characteristic or true of me”), to reflect the level of general anxiety associated with the initiation and maintenance of social interactions [20].

The Kessler Psychological Distress Scale (K10) is a screening instrument used to determine mental illness in health risk appraisal [21]. The K10 comprises 10 questions about emotional states (eg, feelings of fatigue, motor agitation, guilt, restlessness, anxiety, and depression), each of which is rated on a 5-point scale ranging from 1 (“None of the time”) to 5 (“All of the time”) [21]. The individual item scores are summed, yielding an overall score ranging from a minimum of 10 to a maximum of 50, with higher scores indicating higher levels of distress. The K10 scores are categorized as “Low” (10-15), “Moderate” (16-21), “High” (22-29), and “Very high” (30-50) [22].

In addition to these instruments, the health domain also includes questions about injury history and health risk behaviors. Questions about the respondent’s 12-month history of motor vehicle crash incidents, injury due to external trauma, and injury-related hospitalizations have been adapted from the Seguimiento University of Navarra study [10]. Questions about health risk behaviors, which are derived from the New South Wales Population Health Survey [12], consist of questions about smoking (including use of electronic cigarettes), alcohol consumption, illicit and recreational drug use, and sexual behavior.

#### Social Support

The social support domain comprises three subdomains: social connectedness, resilience, and perceived social support. The question about social connectedness and community networks of the respondents is adapted from the Nurses' Health Study II [23]. This question explores how often the respondent takes part in social groups such as workgroups, church-connected groups, self-help groups, charity groups, and public service or community groups. The question about resilience is adapted from the Brief Resilience Scale (BRS) [24]. It includes four items that assess the respondent’s self-reported ability to look for creative ways to alter difficult situations, control reactions, grow in positive ways by dealing with difficult situations, and ways to replace losses encountered in life [24]. Perceived social support is measured using the Multidimensional Scale of Perceived Social Support (MSPSS) [25]. The MSPSS comprises 12 items that are rated on a 7-point Likert scale ranging from “Strongly disagree” to “Strongly agree.” The items are divided into three subscales based on the source of perceived social support (ie, family, friends, and a significant other).

#### Life Events

The Social Readjustment Rating Scale (SRRS) is used to measure the impact of major life events [26]. The SRRS consists of 43 life events considered to be particularly impactful events in the social life of an individual (eg, marriage, death of spouse or a close family member, pregnancy, change in residence, changes in working hours). Each life event has a prespecified weighting (ie, “life units”) based on how traumatic the event felt to the large normative sample. The respondent indicates how many times each life event has occurred during the past 12 months or is expected to occur in the near future. The number of each life event is multiplied by the weights and summed to produce a total score of “life units.”

#### Carer Activities

The questions about carer activities are adapted from the Nurse’s Health Study II [23]. The respondents are asked whether they regularly provide care to a disabled or ill person, and, if applicable, how many hours per week they spend on such carer activities.

#### Social Media and Technology

In regard to the social media and technology domain, the questions are adapted from the Australian 2017 Sensis Social Media Report [27]. The questions focus on the respondent’s use of the internet, use of social networking sites (eg, Facebook, Twitter, and Snapchat), type of devices used to access social networking sites, reasons for using social networking sites, and experiences with using social networking sites.

### Health Record Linkage

Participants are asked to provide consent to have their personal health information retrieved from administrative data collections (ie, ambulance, emergency department, hospital admissions, cancer registry, and mortality records) in New South Wales from January 1, 2017 to 12 months after the baseline survey. Secure data linkage will be conducted by the Centre for Health Record Linkage (CHeReL).

### Data Management

All study information will be obtained, stored, and analyzed in accordance with the National Health and Medical Research Council National Statement on Ethical Conduct in Research Involving Humans [28]. All results will be published in a form that will not allow any individual participants to be identified (ie, in tabular, aggregate form only). The participant registration form contains personal data (eg, name, residential address, email address, mobile phone number, and a relative’s contact details). A participant ID number will be generated for all participants and stored with the data. The participant registration details will be stored separately in a secure password-protected folder. The data collected from the baseline and follow-up surveys will not contain any personally identifiable information, only the participant ID number to allow survey responses to be linked.

For participants who provide consent to have their health records linked to their survey responses, their personal data and participant ID number will be securely transferred to the CHeReL for the purposes of health record linkage. During the record linkage process, the CHeReL will generate a project person number (PPN) for each participant. The CHeReL will not have access to any of the collected data (ie, survey responses or health records). The PPNs are then linked to the existing participant ID numbers in each administrative data collection and returned to the respective data custodians. In turn, the data custodians for each administrative data collection (ie, ambulance, emergency department, hospital admissions, cancer registry, and mortality records) will securely transfer the health data records with PPNs to the researchers. Finally, the researchers will use the PPNs to link survey responses and health records belonging to the same individual, thereby creating a complete data set for analysis.

### Data Analysis

Data will be analyzed using SAS version 9.4 (SAS Institute). The recruitment rate will be calculated as the number of registered participants divided by the total number of Macquarie University graduates in 2018. The denominator data will be supplied by the Macquarie University Graduation Office. The opt-in rates for data linkage of health records and survey responses will be calculated as the number of registered participants opting in divided by the number of registered participants. The representativeness of the sample will be evaluated by comparing its demographic profile with that of the full graduating cohort. Survey drop-out rates will be calculated separately for the baseline and follow-up surveys as the number of participants completing the survey divided by the number of participants starting the survey. Descriptive statistics will be used to evaluate the completeness of the baseline and follow-up surveys. The retention rate of the sample will be calculated as the number of participants completing the follow-up survey divided by the number of participants completing the baseline survey.

## Results

Participant recruitment was completed in October 2018, and data collection for the baseline and follow-up surveys was completed in November 2019. As of April 2020, the process of acquiring health records from administrative data collections has commenced. The findings of this pilot cohort study will be prepared for publication in mid-2020. These findings will include the opt-in and opt-out rates for data linkage of health records and survey responses; a description of the representativeness of recruited participants; a description of the completeness of baseline and follow-up online survey items; and attrition rates for the 12-month follow-up survey.

## Discussion

Emerging adulthood is a unique segment of an individual’s life course. The defining features of this transitional period include identity exploration, instability, possibilities, self-focus, and feeling in-between adolescence and adulthood, all of which are thought to impact quality of life, health, and well-being. A longitudinal cohort study with a comprehensive set of measures would facilitate greater understanding of the multi-faceted elements and unique challenges that contribute to the health and well-being of emerging adults. Before expending significant resources on a large, longitudinal cohort study, it is advisable to first test the feasibility and inform the development of the larger study.

This pilot cohort study aims to evaluate the feasibility of recruiting Australian university graduates to establish a longitudinal cohort study to inform our understanding of emerging adulthood. It will evaluate the ability to recruit university graduates and obtain good quality survey data on a wide range of relevant measures. It is vital to obtain estimates of recruitment, response, and retention (or attrition) rates because these will inform the sample size and statistical power calculations that are necessary for planning and designing a future longitudinal cohort study. An evaluation of the measures in the pilot study is also necessary for the development and selection of the measures to be included in the main study.

There are challenges with recruiting university graduates into cohort studies using web-based surveys. Selection bias is an important consideration as many studies conducted among students report response rates below 20% [29-31]. University students and graduates frequently receive requests to participate in surveys, and this over-surveying can potentially lead to survey fatigue and poor response rates [32]. Compounding the issue is that the average response rate of web surveys is approximately 10% lower than that of mail or telephone surveys [33]. Student engagement, lottery incentives, and extra reminders can be effective for increasing the overall response rate [32,34-36]. However, merely increasing response rate does not necessarily entail diversifying or improving the representativeness of the sample [32]. Although self-selection can lead to unreliable survey outcomes [37], there are potential methods for correcting for selection biases (eg, poststratification or weighting class adjustments, propensity score adjustments, and generalized regression modelling [38]). It has been suggested that student surveys with a 10% or lower response rate can eventually be considered trustworthy if the response quality is good [34].

Response bias is another pervasive problem in the design of surveys. The many types of bias are question design (eg, problems with wording, leading questions, faulty scales, intrusiveness), questionnaire structure (eg, formatting, priming, length, response fatigue), administration of questionnaire (eg, respondent’s learning, recall, primacy or recency depending on mode) [39,40]. Some of these biases can be minimized by adopting previously validated instruments and scales. In the present study, the baseline and follow-up surveys are comprised almost entirely of commonly used and previously validated instruments and scales.

As to limitations, the study is modest in scale, and will be conducted at a single Australian university. This may not be representative of the broader population of university graduates in Australia.

In conclusion, this pilot study comes at a crucial time for research of this kind. It is expected that the findings from the pilot will identify areas for improvement and inform the development of a future longitudinal cohort study.

## Abbreviations

ACTRN: Australian New Zealand Clinical Trials Registry
BRS: Brief Resilience Scale
CHeReL: Centre for Health Record Linkage
EQ-5D: EuroQoL 5-dimension
GAD-7: General Anxiety Disorder scale
HRQOL: health-related quality of life
K10: Kessler Psychological Distress Scale
MQ-MINDS: Macquarie University – Monitoring of Injury and Psychosocial Health Outcomes, Career Trajectories and Continuing Education, Lived Experiences, and Social Connectedness
MSPSS: Multidimensional Scale of Perceived Social Support
RBS: Role Balance Scale
SF-12: Short Form Health Survey
SIAS-6: Social Interaction Anxiety Scale
SRRS: Social Readjustment Rating Scale
